# THE INFLUENCE OF POPULATION AGING ON ENERGY CONSUMPTION QUANTITY MECHANISM RESEARCH

**DOI:** 10.1093/geroni/igad104.2376

**Published:** 2023-12-21

## Abstract

The adjustment of population structure and household size is one of the important factors affecting the amount of household energy consumption. Influenced by the deepening of aging degree and the acceleration of aging development, the amount of energy consumption has also produced a relatively obvious change. Based on the provincial panel data from 2008 to 2017 in China, we used fixed effects and random effects models for regression analysis to further analyze the spatial differences and trends of energy consumption quantity distribution, and explore the mechanism of population aging’s influence on energy consumption quantity. In addition, we explore the path of population aging affecting energy consumption through intermediary effect analysis. Besides, we also analyzes the aging rate of population, energy consumption and environmental protection policies in different countries. It is found that, under the premise of controlling the influence of other variables, population aging rate has a negative impact on the amount of energy consumption, which is different between urban and rural areas. The increase of population aging rate in urban areas may lead to the increase of energy consumption, while the increase of population aging rate in rural areas may lead to the decrease of energy consumption. Household size and household consumption level are important intermediary variables between population aging and energy consumption. Population aging can indirectly affect energy consumption through affecting household size and household consumption level.

